# POLICY SERIES: CONGRESSIONAL UPDATE

**DOI:** 10.1093/geroni/igz038.2000

**Published:** 2019-11-08

**Authors:** Brian W Lindberg

**Affiliations:** The Gerontological Society of America, Washington, District of Columbia, United States

## Abstract

This popular annual session will provide cutting-edge information on what the 116th Congress has and has not accomplished to date, and what may be left for this year. Speakers will discuss key issues such as Social Security, Medicare, Medicaid, and the Older Americans Act, caregiving, the National Institutes of Health. Hill staffers, advocates, and lobbyists will present.

